# Nurses and Physicians in a Medical Admission Unit Can Accurately Predict Mortality of Acutely Admitted Patients: A Prospective Cohort Study

**DOI:** 10.1371/journal.pone.0101739

**Published:** 2014-07-14

**Authors:** Mikkel Brabrand, Jesper Hallas, Torben Knudsen

**Affiliations:** 1 Department of Medicine, Hospital of South West Denmark, Esbjerg, Denmark; 2 Centre South Western Denmark, Institute of Regional Health Research – University of Southern Denmark, Esbjerg, Denmark; 3 Research Unit of Clinical Pharmacology, University of Southern Denmark, Odense, Denmark; San Raffaele Scientific Institute, Italy

## Abstract

**Background:**

There exist several risk stratification systems for predicting mortality of emergency patients. However, some are complex in clinical use and others have been developed using suboptimal methodology. The objective was to evaluate the capability of the staff at a medical admission unit (MAU) to use clinical intuition to predict in-hospital mortality of acutely admitted patients.

**Methods:**

This is an observational prospective cohort study of adult patients (15 years or older) admitted to a MAU at a regional teaching hospital. The nursing staff and physicians predicted in-hospital mortality upon the patients' arrival. We calculated discriminatory power as the area under the receiver-operating-characteristic curve (AUROC) and accuracy of prediction (calibration) by Hosmer-Lemeshow goodness-of-fit test.

**Results:**

We had a total of 2,848 admissions (2,463 patients). 89 (3.1%) died while admitted. The nursing staff assessed 2,404 admissions and predicted mortality in 1,820 (63.9%). AUROC was 0.823 (95% CI: 0.762–0.884) and calibration poor. Physicians assessed 738 admissions and predicted mortality in 734 (25.8% of all admissions). AUROC was 0.761 (95% CI: 0.657–0.864) and calibration poor. AUROC and calibration increased with experience. When nursing staff and physicians were in agreement (±5%), discriminatory power was very high, 0.898 (95% CI: 0.773–1.000), and calibration almost perfect. Combining an objective risk prediction score with staff predictions added very little.

**Conclusions:**

Using only clinical intuition, staff in a medical admission unit has a good ability to identify patients at increased risk of dying while admitted. When nursing staff and physicians agreed on their prediction, discriminatory power and calibration were excellent.

## Introduction

Prognostication is an essential component of many decisions made by physicians, including choices between treatment options and dispositions (eg, between a floor or ICU admission), and about attempting resuscitation. But prognostication is no longer taught systematically and can be seen as a forgotten art of medicine.[1] Several risk stratification systems of varying complexity have been developed for use in the emergency and admission environment.[2] However, it is possible that staff in a medical admission unit can provide equally good—or even better—predictions using mainly their clinical intuition, thus rendering the use of complex risk stratification tools unnecessary.

Surprisingly, this possibility has not yet been well studied in emergency departments and admission units. To our knowledge, only one study has examined the ability of staff in a medical admission unit to prognosticate patients at admission, and from the few other studies that actually touch upon this subject, no clear conclusions can be drawn.[3], [4] In a 1986 study Charlson et al., asked residents in a medical admission unit in New York, USA, to assess the level of severity of 604 acutely medical patients. They found the assessment by the physicians to be an accurate estimate of mortality[5]. The subject has been better examined in other settings. In the only meta-analysis on this subject from the critical care environment (a 2006 systematic review of 12 studies), Sinuff and colleagues[6] found that ICU physicians (regardless of experience) were significantly better than scoring systems at identifying at-risk patients.

As prognostication is an integral part of modern medicine, we designed the present study to evaluate the capability of the medical admission unit to accurately predict the in-hospital mortality of acutely admitted medical patients.

## Materials and Methods

### Setting

The present study was part of a larger (aimed at developing a novel and validating existing risk prediction scores) prospective observational cohort study of all patients who were acutely admitted to a medical admission unit (MAU) at Sydvestjysk Sygehus Esbjerg, a Danish 460-bed regional teaching hospital with a contingency population of 220000. This hospital includes all sub-specialties of internal medicine, as well as paediatrics, general surgery, orthopaedic surgery, and a 12-bed intensive care unit. Nurses, nurse assistants, interns, residents, and attending physicians staff the department around the clock.

This study included all patients ≥15 years of age who were acutely admitted from 23 February to 26 May 2010. There were no exclusion criteria and patients were included in calculations if the required data were available. Patients could be admitted from the emergency department, outpatient clinics, by the ambulance service, the out-of-hours emergency medical service, or by their family physician. When a patient arrived in the MAU, they were administratively admitted to the hospital and could later be transferred to other departments or discharged home from the MAU.

### Data collection

For each patient, the first nurse or nurse assistant (a trained health care professional with approx. two years of training) to see the patient recorded vital signs, demographic information, and their subjective probability of in-hospital mortality (as a number between 0 and 100%). The first physician to see the patient was also asked to report their subjective probability of in-hospital mortality (a number between 0 and 100%). Predictions were to be made immediately upon their first assessment of the patient, without waiting for initial test results, or other information. However, vital signs, past medical history, and current medication most often would have been available at the time of prediction for both members of the nursing staff and physicians.

To enable the comparison of the staff predictions to results from an established objective system, we also calculated the Worthing physiological score (WPS) for each patient. WPS was developed and validated in an admission unit similar to ours, and also uses in-hospital mortality as the endpoint.[7], [8] The score calculation required systolic blood pressure, pulse, respiratory rate, temperature, peripheral oxygen saturation in room air, and level of consciousness (defined as alert, responsive to vocal stimuli, responsive to pain, or unresponsive). Other existing prediction systems all use other endpoints making comparison with staff assessment inappropriate.

After all patients were enrolled, in-hospital mortality data were extracted from the Danish Person Register[9] and the Danish National Registry of Patients.[10] All Denmark residents have a personal identification number, allowing cross-linking across both local and national databases. The Danish Person Register contains the vital status of all persons with occupancy in Denmark,[9] while the Danish National Registry of Patients tracks all contacts with the health care system.[10] We were thus able to have complete follow-up information for all patients. Danish law does not require approval by the regional ethics committee for observational studies.

### Statistics

The sample size was dictated by other parts of the larger study. Briefly, the entire study was powered to develop and validate a risk stratification system predicting seven-day all-cause mortality. The cohort for the present study was collected as a validation cohort.

To assess the ability of each system to discriminate between survivors and non-survivors (ie, the discriminatory power), we calculated the area under the receiver-operating characteristics curve (AUROC). AUROC is a summary measure of sensitivity and specificity at each possible cut-off, essentially representing the probability that a patient who dies while admitted will have a higher score than a patient who survives. An AUROC above 0.8 represents excellent discriminatory power.[11]

The accuracy of predictions, how close are the predictions to the actual outcome (ie, the calibration), was assessed using the Hosmer-Lemeshow goodness-of-fit test,[11] which assesses whether the observed event rate matches the expected event rate. A p-value above 0.05 indicates acceptable calibration.[11] Calibration of the WPS system was assessed according to the method of Seymour et al.[12] Briefly, we predicted the probabilities of the individual scores using logistic regression analysis, the population was divided into decentiles by expected event rate, and we calculated the Hosmer-Lemeshow goodness-of-fit test.

To test the sensitivity of the WPS, we also calculated the Prytherch score[13] for all patients. This biochemical score was calculated using gender, mode of admission, age, urea, sodium, potassium, albumin, haemoglobin, white cell count, creatinine, and urea/creatinine ratio.[13] Also, to test if the addition of the WPS or Prytherch scores to the assessment made by the nursing staff and physicians, we calculated the integrated discrimination improvement[14].

The present study is reported in accordance with the STROBE statement.[15] All data are presented descriptively, as median and inter-quartile range (IQR) or proportions (with 95% confidence interval [CI]) as appropriate. Differences between groups were tested using Wilcoxon Rank Sum test or χ^2^-test. Comparisons of discriminatory power among nursing staff predictions, physician predictions, and WPS were performed following the method introduced by Hanley and McNeil.[16] Stata 12.1 (Stata Corp, College Station, TX, USA) was used for analyses.

## Results

A total of 2848 admissions (2463 individual patients, 276 patients had more than one admission) were included in our study; see table 1. All admissions were considered individual events and included in the study. The nursing staff had a median of 7.4 (2.9–14.0) years of experience, and the physicians had a median of 3.8 (1.7–7.2) years.

**Table 1:** Characteristics of admissions.

Variable	Total, n = 2848	Died during admission, n = 89	Survived admission, n = 2759
Female, n (%)	1490 (52.3%)	43 (48.3%)	1447 (52.5%)
Age, years (inter-quartile range)	64 (48–76)	78 (70–84)	64 (48–76)
Seven-day mortality, n (%)	57 (2.0%)	51 (57.3%)	6 (0.2%)
In-hospital mortality, n (%)	89 (3.1%)	-	-
Length of stay, days	1 (1–5)	5 (2–14)	1 (1–5)

### Predictions by nursing staff

**Figure 1:**
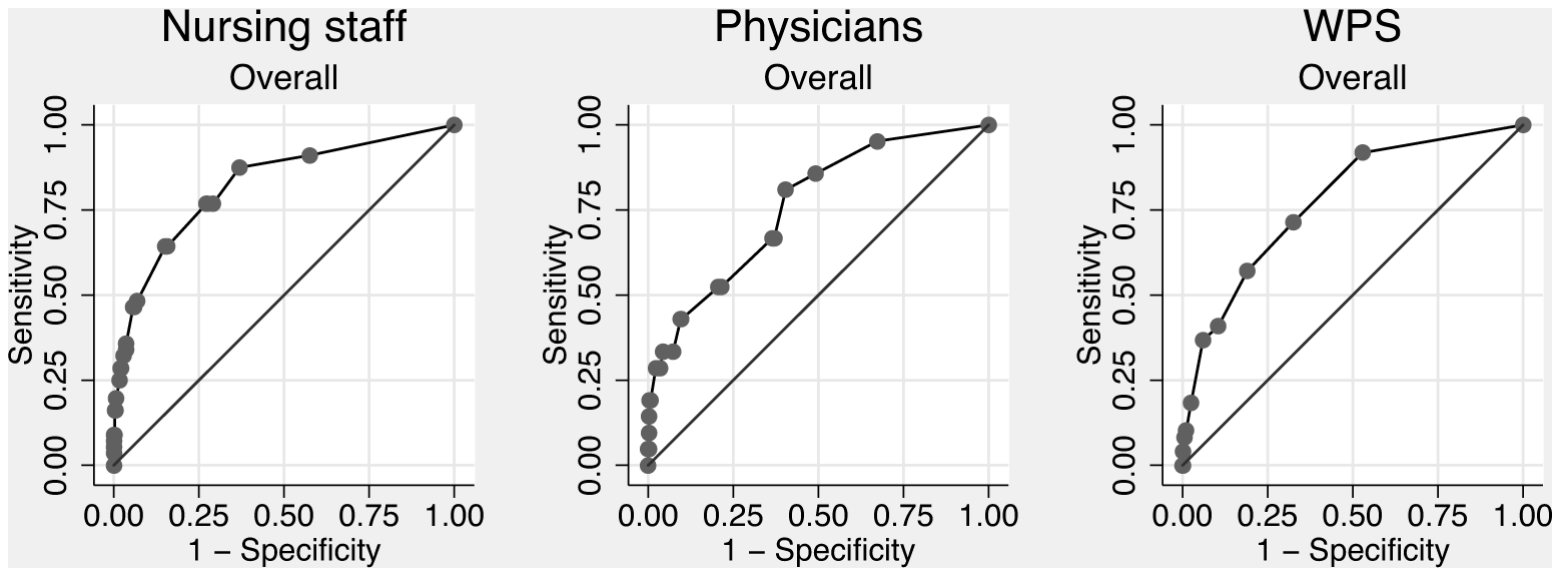
ROC curves of overall predictions. WPS = Worthing physiological scoring system.

**Figure 2:**
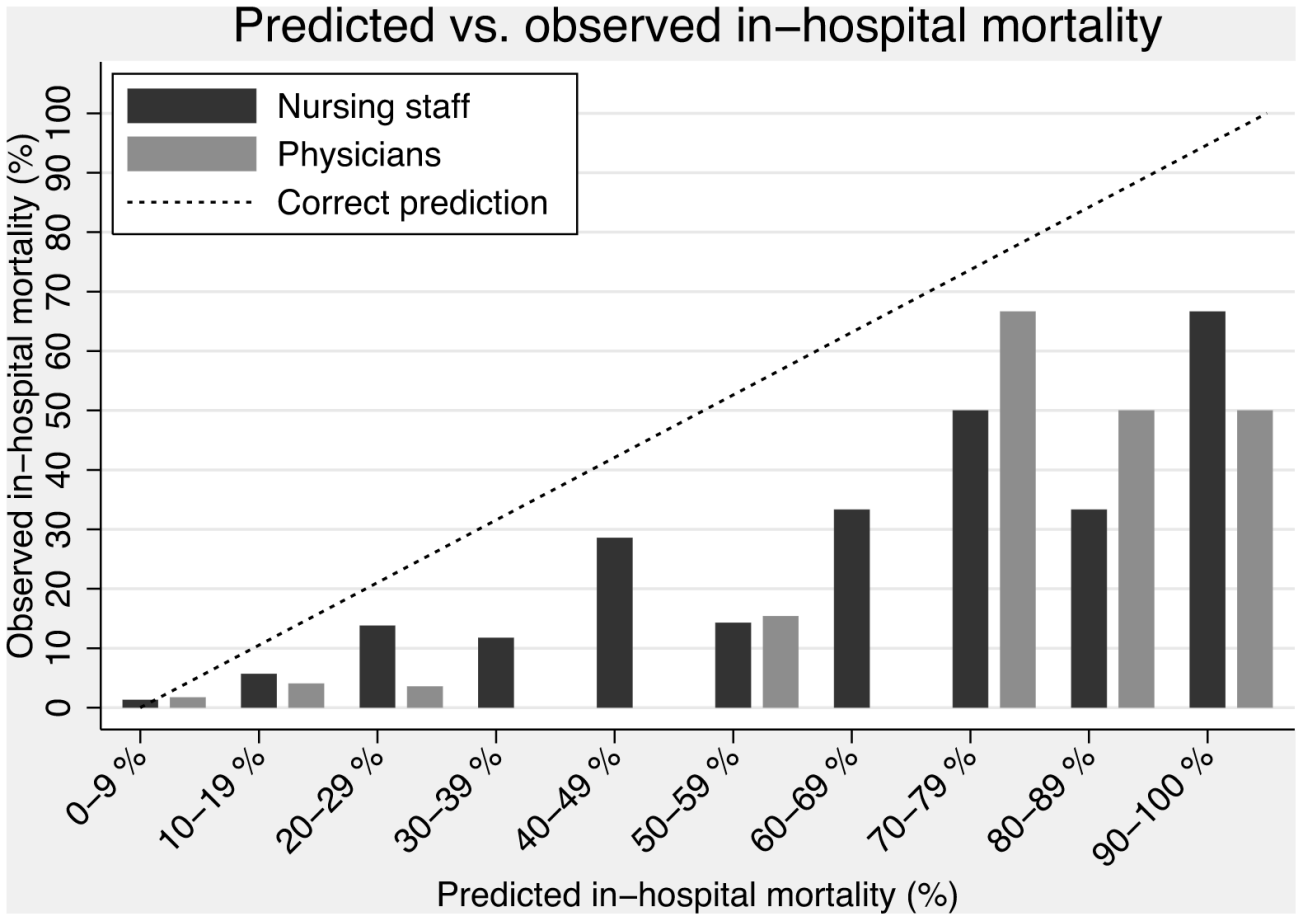
Predicted vs. observed in-hospital mortality.

**Figure 3:**
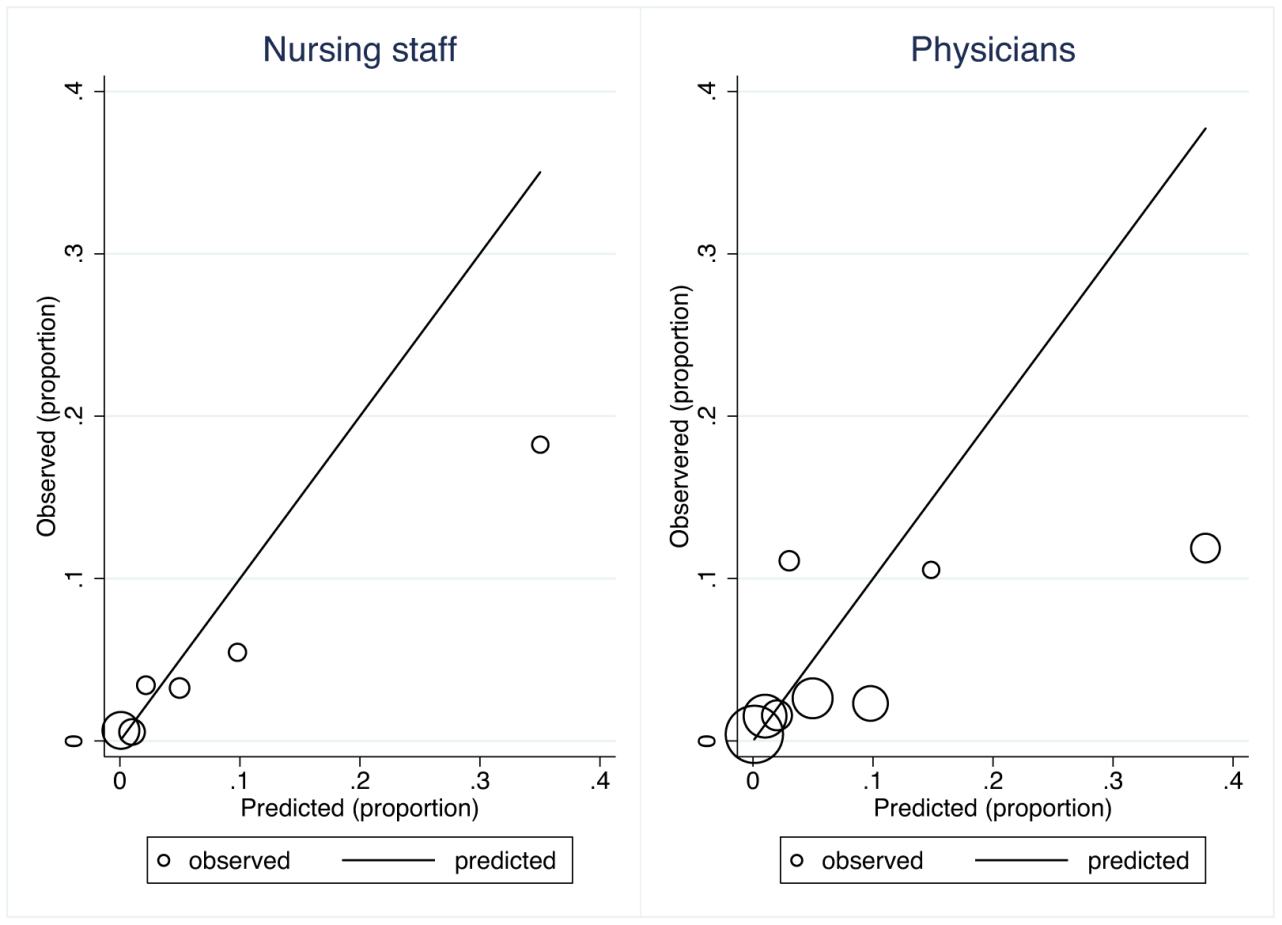
Calibration plot for nursing staff and physicians.

The nursing staff (nurses and nurse assistants) completed forms on 2404 (84.4%) admissions, and a prediction of in-hospital mortality was made in 1820 (63.9% of all admissions). The overall discriminatory power of the nursing staff was 0.823 (95% CI: 0.762–0.884) (figure 1). Calibration failed, as the Hosmer-Lemeshow goodness-of-fit χ^2^-test result was 49.2 (4 degrees of freedom), p<0.0001 (figure 2 and figure 3). Discriminatory power increased with increasing experience, but calibration failed unless the nurse had more than 10 years of experience (table 2). Nurses alone (excluding nursing assistants) assessed 1499 patients with an overall discriminatory power of 0.839 (95% CI: 0.778–0.900), while nurse assistants (excluding nurses) assessed 248 patients with an overall discriminatory power of 0.885 (95% CI: 0.797–0.973). Calibration failed for both groups.

**Table 2:** Discriminatory power (95% confidence interval) and calibration of nursing staff and physician predictions stratified by experience.

Experience	Discriminatory power				Calibration				Fatalities/Admissions		
	Nursing staff	Physicians	All	WPS	Nursing staff	Physicians	All	WPS	Nursing staff	Physicians	WPS
Overall	0.823 (0.762–0.884)	0.761 (0.657–0.864)	0.808 (0.756–0.859)	0.776 (0.713–0.840)	p<0.0001	p<0.0001	p<0.001	p = 0.0990	56/1820	21/734	49/1999
<5 years	0.728 (0.587–0.869)	0.748 (0.628–0.868)	0.739 (0.648–0.831)	-	p<0.0001	p = 0.0002	p<0.001	-	16/609	16/555	-
5–9 years	0.774 (0.615–0.932)	0.955 (0.915–0.994)	0.774 (0.627–0.921)	-	p = 0.0024	p = 0.2474	p<0.01	-	10/334	1/111	-
10–14 years	0.886 (0.791–0.982)	0.739 (0.562–0.917)	0.877 (0.784–0.969)	-	p = 0.1273	p = 0.2072	p = 0.38	-	17/363	1/24	-
≥15 years	0.874 (0.797–0.950)	0.846 (0.540–1.000)	0.870 (0.791–0.950)	-	p = 0.0346	p = 0.0702	p = 0.13	-	11/503	3/43	-

WPS = Worthing physiological score.

### Predictions by physicians

The doctors completed forms on 738 (25.9%) admissions, and a prediction of in-hospital mortality was made in 734 (25.8% of all admissions). The overall discriminatory power of the physicians was 0.761 (95% CI: 0.657–0.864) (figure 1). Calibration failed, as the Hosmer-Lemeshow goodness-of-fit χ^2^-test result was 32.8 (6 degrees of freedom), p<0.0001 (figure 2 and figure 3). As with the nursing staff, discriminatory power increased with experience, but this finding must be interpreted cautiously due to the small sample size (table 2). In contrast to our findings with the nursing staff, calibration only failed for the most inexperienced physicians, while doctors with ≥5 years of experience had acceptable calibration (table 2).

### Patients with agreement between nurse and physician assessment

Of the 507 admissions assessed by both nursing staff and physicians, there was agreement (within ±5%) on 385 (75.9%). In these cases with agreement, the overall AUROC was 0.898 (95% CI: 0.773–1.000) and calibration was almost perfect with a Hosmer-Lemeshow goodness-of-fit result of 3.34 (8 degrees of freedom), p = 0.91. In cases with disagreement, the overall AUROC was 0.732 (95% CI: 0.595–0.868) and calibration was acceptable. When inexperienced physicians (<5 years) agreed with experienced nurses (≥5 years) (n = 191), AUROC was 0.820 (95% CI: 0.565–1.00), with almost perfect Hosmer-Lemeshow goodness-of-fit test of 1.98 (8 d.f.), p = 0.98. If both were inexperienced, i.e. <5 years (n = 89), AUROC was 0.818 (95% CI, 0.735–0.902) and Hosmer-Lemeshow goodness-of-fit 2.65 (8 d.f.), p = 0.92.

### Predictions as a result of experience

If we combined all predictions made by physicians, nurses and nursing assistants, we had a total of 2,554 predictions. As seen in table 2, the discriminatory power and calibration increased with increasing experience, but as the nursing staff made most predictions, the AUROCs and goodness of fit tests deviated little from their results.

### Worthing physiological scoring system

WPS could be calculated for 1999 admissions (70.2%). The discriminatory power was 0.776 (95% CI: 0.713–0.840) (figure 1 and table 2). Calibration was acceptable, as the Hosmer-Lemeshow goodness-of-fit χ^2^-test result was 7.8 (4 degrees of freedom), p = 0.10.

### Combination of staff prediction and Worthing physiological score

We applied the original definition of WPS[7] and combined it with the predictions by the nursing staff (grouped into low risk [0–4%], intermediate risk [5–9%], and high risk [10–100%]). When the nurse found the patient to be at low or intermediate risk, this held true regardless of WPS (table 3). However, if the nurse found the patient to be at high risk, there was an incremental rise in mortality with increasing WPS — higher than that indicated by WPS alone (table 3). This pattern was not observed when combining WPS and physician estimates (table 3).

**Table 3:** In-hospital mortality stratified by Worthing physiological score (WPS) and predictions by nursing staff and physicians.

	WPS alone	Nursing staff			Physicians		
WPS		Low risk (0–4%)	Intermediate risk (5–9%)	High risk (10–100%)	Low risk (0–4%)	Intermediate risk (5–9%)	High risk (10–100%)
Low risk (score 0–1)	12/939 (1.3%)	6/757 (0.8%)	3/96 (3.1%)	3/86 (3.5%)	2/248 (0.8%)	1/48 (2.1%)	1/45 (2.2%)
Intermediate risk (score 2–6)	17/447 (3.8%)	6/313 (1.9%)	2/62 (3.2%)	9/72 (12.5%)	2/92 (2.2%)	2/31 (6.5%)	3/48 (6.3%)
High risk (score 7–14)	3/17 (17.7%)	0/1 (0.0%)	0/2 (0.0%)	3/14 (21.4%)	1/2 (50.0%)	0/1 (0.0%)	1/4 (25.0%)

The numbers indicate the number of deaths/total number of admissions in the strata (%).

### Comparison of predictions

We compared the discriminatory power of predictions made by the nursing staff, physicians, and WPS, as well as when the nursing staff and physicians were in agreement (within ±5%). Such comparisons were only possible for the admissions that were assessed by all of the groups being compared. These comparisons indicated that physician predictions (AUROC 0.764, 95% CI: 0.659–0.868) were worse than predictions for which the nursing staff and physicians were in agreement (AUROC 0.892, 95% CI: 0.826–0.958), p = 0.02.

### Sensitivity analysis

Substituting the WPS with the Prytherch score[13] did not lead to significantly different results (data not shown) in the scores, or for analyses of combinations of staff predictions and scores. Calculating the integrated discrimination improvement for both WPS and the Prytherch score only showed significant improvement when adding the Prytherch score to the predictions made by the nursing staff (p = 0.002).

### Selection bias

As both nursing staff and physicians failed to predict in-hospital mortality for all patients, there is a risk of selection bias. However, we found no indication of this (table 4).

**Table 4:** Indicators of potential selection bias stratified by staff group.

	Nursing staff			Physicians		
Indicator	Assessed admissions, n = 1820	Non-assessed admissions, n = 1028	p-value	Assessed admissions, n = 734	Non-assessed admissions, n = 2114	p-value
Age, years	65 (50–76)	64 (46–76)	0.07	63 (48–75)	65 (48–76)	0.16
In-hospital mortality, n (%)	56 (3.1%)	33 (3.2%)	0.96	21 (2.9%)	68 (3.2%)	0.68
WPS score	1 (0–2)	1 (0–2)	0.40	1 (0–2)	1 (0–2)	0.65
Predicted mortality by Prytherch score, %	8.0% (3.1–17.1%)	7.3% (2.7–16.9%)	0.17	7.4% (3.0–16.2%)	8.0% (2.9–17.3%)	0.37
Length of stay, days	1 (1–5)	1 (1–5)	0.49	1 (1–5)	1 (1–5)	0.80
Charlson co-mobidity score	2 (1–4)	2 (1–4)	0.61	2 (1–4)	2 (1–4)	0.03

Data is reported as median (inter-quartile range) unless otherwise specified. WPS = Worthing physiological score. IQR = inter-quartile range.

## Discussion

Overall, our results indicated that the nursing staff overall was excellent at predicting in-hospital mortality of acutely admitted medical patients. However, their precision was poor, as calibration failed except with the most experienced nurses. Physicians were no better than the nurses at predicting in-hospital mortality but their predictions improved with 5 years of experience. Compared to an objective risk stratification system, the nursing staff and the physicians were not better or worse at predicting in-hospital mortality. A combination of staff prediction and an objective scoring system resulted in little improvement.

Mortality prediction by staff in a medical admission unit has only once previously been studied in a broad group of patients in an emergency or admission setting. However, in the previously mentioned study by Charlson et al., the statistical analyses were not focused on the discrimination or calibration and thus not directly comparable[5]. In the only other comparable previous study, Buurman and colleagues[3] asked attending nurses and physicians to give their clinical impression of the illnesses of 463 acutely admitted elderly medical patients. They combined the clinical impression for bad outcome (including mortality) with a score based on functional impairment, a malignant diagnosis, co-morbidity, and high blood urea nitrogen level, and found that this did not improve the discriminatory power. In contrast, we found a synergistic effect when we combined nursing staff prediction with WPS.

The nursing staff was excellent at identifying patients at an overall increased risk of dying. However, their precision (calibration) was poor, and their predictions were generally too optimistic. This phenomenon has been previously shown in various other settings.[17]–[19] However, a well-performed meta-analysis on physician prediction in the ICU setting showed that physician predictions of mortality were more accurate than scoring systems.[6] Looking at patients at high risk (estimated risk of 10% or more by the staff), table 3 shows that both the nursing staff and physicians had difficulty identifying these patients. Of 172 patients identified by the nursing staff as being at increased risk, only 15 (8.7%) died, while the physicians identified 97 patients to be at increased risk and only 5 (5.2%) died. Combining the staff prediction with an objective score (ie, WPS) improved the identification, but the results were still not impressive. As a consequence, we have to continue to improve our tools to identify patients at increase risk of dying. One advantage of using established scores, like WPS or MEWS, is that they are generally well known and that most health care staff understands them and thus know what is meant by a patient with a score of (eg) three. When using clinical intuition, there is no standardised, uniformly understood output and this limits their use. We found, that for some patients, nursing staff outperformed physicians in prognostication. We can only speculate to why this is, but the nurses were far more experienced that the physicians. Also, our nursing staff is only employed in the medical admission unit, and are thus confronted regularly with sick patients, whereas most of our physician have other functions in the hospital (e.g. out-patient clinic, endoscopy etc.). This result in the nurses quickly gaining the experience physicians requires longer time to accumulate.

Although clinicians are constantly faced with making decisions based on prognosis, there has been a longstanding uncertainty regarding the best basis for these decisions. The Ethics Committee of the Society of Critical Care Medicine has argued against using scoring systems to predict outcome for individual patients[20] but, as we have shown, the alternative (ie, clinical intuition) is not more accurate. Making irreversible decisions on individual patient care, eg withdrawal of care, has to be based on systems with very high specificity and, from our data, one could fear that staff prediction alone are not sufficient. We found little effect of combining WPS with staff prediction; however, when the nursing staff and physicians agreed, discriminatory power was 0.898 and calibration was almost perfect. Other decisions in health care, eg identification of patients requiring increased aggressive treatment, require systems with a high sensitivity. Our data suggests that perhaps staff assessment alone can be used in this context. However, use of clinical assessment by staff holds a risk of being a self-fulfilling prophecy. It is possible that a patient perceived to be at high could receive inferior treatment than a patient believed to be at better health.

Our study has several limitations that need to be addressed. First, physicians assessed a much smaller percentage of patients, compared to that assessed by the nursing staff. This difference could have led to some selection bias, although we found no statistical evidence of this. The small percentage of physician-assessed patients also limits the validity of our results, as some calculations were based on very small numbers, resulting in wide confidence intervals and potential bias. Should this study be repeated, the staff should be better compelled to complete the forms and assess the patients. Second, we did not study how the staff made their predictions; they may have used parts of existing prediction systems, co-morbidities, or the predictive nature of abnormal vital signs. We were unable to completely blind the staff to any of this information, which could affect our results. Third, we did not assess inter- or intra-rater reliability, as each patient was assessed by only one nurse and one physician. Fourth, most of the physicians in our study were inexperienced, with a median experience of 3.8 years; many other emergency departments and admission units will be staffed by more experienced physicians. Fifth, our study is a single centre study from the MAU at a teaching hospital in Denmark. This setting is not uniform to the rest of the world, and this limits the generalizability of our findings. Finally, our choice of in-hospital mortality as the predicted endpoint may have influenced the results. By nature, in-hospital mortality is during a period of varying length and possibly extensively long. Had we chosen a set time (eg, one-week mortality) our results could have been different.

## Conclusions

Our study of predictions of the in-hospital mortality of acutely admitted medical patients by staff in a medical admission unit, showed that nursing staff predictions were insignificantly better than those of physicians and an objective scoring system. The predictive ability of staff increased with experience. The combination of staff prediction and objective scoring system did not significantly improve the predictive power. Patients identified as being at low and intermediate risk by the nursing staff, had low mortality regardless of their objective score, while patients designated as being at high risk had increasing mortality with increasing score. Our findings indicate that the in-hospital mortality predictions of experienced staff at patient admission have prognostic value similar to that of validated objective scoring systems.
